# Impact of the number of resected lymph nodes on survival after preoperative radiotherapy for esophageal cancer

**DOI:** 10.18632/oncotarget.8113

**Published:** 2016-03-16

**Authors:** San-Gang Wu, Zhao-Qiang Zhang, Wen-Ming Liu, Zhen-Yu He, Feng-Yan Li, Huan-Xin Lin, Jia-Yuan Sun, Hui Lin, Qun Li

**Affiliations:** ^1^ Department of Radiation Oncology, the First Affiliated Hospital of Xiamen University, Xiamen 361003, People's Republic of China; ^2^ Eye Institute of Xiamen University, Fujian Provincial Key Laboratory of Ophthalmology and Visual Science, Medical College of Xiamen University, Xiamen 361003, People's Republic of China; ^3^ Department of Gastroenterology, Zhongshan Hospital of Xiamen University, Xiamen 361003, People's Republic of China; ^4^ Sun Yat-sen University Cancer Center, State Key Laboratory of Oncology in South China, Department of Radiation Oncology, Collaborative Innovation Center of Cancer Medicine, Guangzhou 510060, People's Republic of China; ^5^ Department of Cardiovascular and Thoracic Surgery, The People's Hospital of Guangxi Zhuang Autonomous Region, Nanning 530021, People's Republic of China

**Keywords:** esophageal cancer, lymph nodes, preoperative radiotherapy, survival, SEER

## Abstract

To assess the impact of the number of resected lymph nodes (RLNs) for survival in esophageal cancer (EC) patients treated with preoperative radiotherapy and cancer-directed surgery. The Surveillance Epidemiology and End Results (SEER) database was queried to identify EC patients treated from 1988 to 2012 who had complete data on the number of positive lymph nodes and number of RLNs. Kaplan–Meier survival analysis and Cox regression proportional hazard methods were used to determine factors that significantly impact cause-specific survival (CSS) and overall survival (OS). There were a total of 3,159 patients who received preoperative radiotherapy and cancer-directed surgery. The median number of RLNs was 10 in both patients who received and did not receive preoperative radiotherapy (*P* = 0.332). Cox regression univariate and multivariate analysis showed that RLN count was a significant prognostic factor for CSS and OS. Patients with 11–71 RLNs had better CSS (hazard ratio [HR] = 0.694, 95% confidence interval [CI]: 0.603–0.799, *P* < 0.001) and OS (HR = 0.724, 95% CI: 0.636–0.824, *P* < 0.001) than patients with 1–10 RLNs. The 5-year CSS rates were 39.1% and 44.8% in patients with 1–10 RLNs and 11–71 RLNs, respectively (*P* < 0.001). The 5-year OS rates were 33.7% and 39.9% in patients with 1–10 RLNs and 11–71 RLNs, respectively (*P* < 0.001). A higher number of RLNs was associated with better survival by tumor stage and nodal stage (all *P* < 0.05). RLN count is an independent prognostic factor in EC patients who undergo preoperative radiotherapy and cancer-directed surgery.

## INTRODUCTION

The randomized Chemoradiotherapy for Oesophageal Cancer Followed by Surgery Study (CROSS) revealed the efficacy of neoadjuvant chemoradiotherapy (nCRT) combined with surgery was superior to that of surgery alone [1, 2]. Currently, nCRT combined with surgery is the major therapeutic strategy for locally advanced esophageal cancer (EC) [1, 2]. Additionally, studies have indicated that nCRT affects the mode of EC recurrence, the recurrence rate of mediastinal lymph nodes is significantly lower in patients who receive nCRT combined with surgery than in patients who receive surgery only [3, 4]. Even though the CROSS study found that the number of resected lymph nodes (RLNs) had no influence on survival of EC patients [5], the prognostic and therapeutic value of lymphadenectomy in EC patients who receive neoadjuvant therapy remains controversial [6–9]. RLN count is the main criteria for evaluating the completeness of lymphadenectomy. If lymph nodes are not completely resected, the accuracy of staging is affected and the risk of tumor recurrence is increased due to remaining potentially positive lymph nodes.

In an earlier Surveillance, Epidemiology, and End Results (SEER) study, it was reported that preoperative radiotherapy was an independently prognostic factor for survival in EC patients [10]. A meta-analysis revealed that preoperative chemotherapy had no influence on the survival of EC patients [11]. However, another study indicated that nCRT could improve the survival of patients with locally advanced EC [12]. Preoperative radiotherapy has clinical significance for EC in terms of being a regional treatment. However, it is worthy to note that nCRT has an effect on the number of RLNs [5, 13], which could potentially affect the prognosis. Therefore, in this study we investigate the prognostic value of the number of RLNs in the EC patients who received preoperative radiotherapy using a population-based analysis of the SEER database.

## RESULTS

### Patient characteristics and lymph node resection

Over the study period, 3,159 patients with EC who received preoperative radiotherapy and cancer-directed surgery were identified, and their clinical characteristics are shown in Table 1. The median age of the patients was 62 years (range, 20–87 years), and 84.1% were male. There were 2,141 patients (67.8%) with esophageal adenocarcinoma, and 720 (22.8%) with squamous cell carcinoma. Of the 3,089 patients whose tumor location was available, 2,594 tumors (84.0%) were located in the lower esophagus. The median number of RLNs was 10 (25th percentile 6, 75th percentile 17; range, 1–71) in patients who receive preoperative radiotherapy, and 10 in patients without preoperative radiotherapy (*n* = 5,805, *P* = 0.332). Overall, 2,039 patients (64.5%) had node-negative disease and 1,120 (35.5%) had nodal metastases. In patients with nodal metastases, the median number of involved lymph nodes was 2 (range, 1–24) and the median LNR was 0.20 (range, 0.02–1.0).

**Table 1:** Baseline characteristics of patients with esophageal cancer

Characteristic	*n*	1–6 RLNs (%)	7–10 RLNs (%)	11–17 RLNs (%)	18–71 RLNs (%)	*P* value
Year of diagnosis						
1988–1994	47	28 (2.9)	8 (1.2)	9 (1.1)	2 (0.2)	< 0.001
1995–1999	193	87 (9.1)	45 (6.7)	41 (5.1)	20 (2.8)	
2000–2004	826	326 (34.0)	199 (29.4)	185 (22.9)	116 (16.2)	
2005–2012	2093	519 (54.0)	423 (62.7)	572 (70.9)	579 (80.8)	
Race						
Black	176	60 (6.3)	46 (6.8)	36 (4.5)	34 (4.7)	0.279
White	109	33 (3.4)	18 (2.7)	29 (3.6)	29 (4.1)	
Other/unknown	2874	867 (90.3)	611 (90.5)	742 (91.9)	654 (91.2)	
Age (years)						
< 60	1338	404 (42.1)	301 (44.6)	356 (44.1)	277 (38.6)	0.090
≥ 60	1821	556 (57.9)	374 (55.4)	451 (55.9)	440 (61.4)	
Sex						
Male	2656	801 (83.4)	563 (83.4)	674 (83.5)	618 (86.2)	0.376
Female	503	159 (16.6)	112 (16.6)	133 (16.5)	99 (13.8)	
Histologic subtype						
Squamous	720	255 (26.6)	154 (22.8)	160 (19.8)	151 (21.1)	0.029
Adenocarcinoma	2141	617 (64.3)	454 (67.3)	575 (71.3)	495 (69.0)	
Other	298	88 (9.1)	67 (9.9)	72 (8.9)	71 (9.9)	
Tumor location (*n* = 3089)						
Upper third	56	24 (2.6)	7 (1.1)	9 (1.1)	16 (2.3)	< 0.001
Middle third	439	168 (17.7)	88 (13.4)	83 (10.6)	100 (14.3)	
Lower third	2594	756 (79.7)	562 (85.5)	695 (88.3)	581 (83.4)	
Tumor stage (*n* = 2263)						
T1	357	121 (20.8)	81 (17.5)	78 (12.6)	77 (12.8)	0.001
T2	408	110 (18.9)	90 (19.4)	101 (16.4)	107 (17.8)	
T3	1358	319 (54.8)	269 (58.0)	391 (63.4)	379 (63.0)	
T4	140	32 (5.5)	24 (5.1)	47 (7.6)	39 (6.4)	
Nodal stage						
N0	2039	697 (72.6)	420 (62.2)	490 (60.7)	432 (60.3)	< 0.001
N1	715	200 (20.8)	171 (25.3)	185 (22.9)	159 (22.1)	
N2	308	63 (6.6)	72 (10.7)	94 (11.7)	79 (11.0)	
N3	97	0 (0)	12 (1.8)	38 (4.7)	47 (6.6)	
Grade (*n* = 2789)						
Well differentiated	137	44 (5.2)	24 (4.1)	33 (4.7)	36 (5.5)	0.940
Moderately differentiated	1169	348 (41.2)	249 (42.4)	298 (42.4)	274 (41.9)	
Poorly/undifferentiated	1483	453 (53.6)	314 (53.5)	372 (52.9)	344 (52.6)	

RLNs, resected lymph nodes.

Given that RLN count was a continuous variable, the numbers of RLNs were examined as categorical variables based on quartiles. Patients were divided into quartiles according to their RLNs counts (Group 1 [1–6, *n* = 960], Group 2 [7–10, *n* = 675], Group 3 [11–17, *n* = 807], and Group 4 [18–71, *n* = 717]).

RLN count was associated with the year of diagnosis (*P* < 0.001), histological type (*P* = 0.029), tumor location (*P* < 0.001), tumor (T) stage (*P* = 0.001), and nodal (N) stage (*P* < 0.001), but was not associated with age, race, sex, and grade (all *P* > 0.05) (Table 1).

### Analysis of prognosis

Cox regression univariate analysis showed that year of diagnosis, age, sex, T stage, N stage, grade, LNR (continuous variable), and RLN count as a continuous variable or as a categorical variable were significant prognostic factors for CSS and OS (all *P* < 0.05) (Table 2). Subgroup analysis showed no significant difference between Group 1 and 2 with respect to CSS (*P* = 0.502) and OS (*P* = 0.727), or between Group 3 and 4 with respect to CSS (*P* = 0.090) and OS (*P* = 0.084), and the CSS (*P* = 0.013) and OS (*P* = 0.032) were significant difference between Group 2 and 3. Thus, for further analysis, the Group 1 and 2 (1–10 RLNs) were combined, and Group 3 and 4 (11–71 RLNs) were combined.

**Table 2:** Univariate analysis of prognostic factors influencing the survival of esophageal cancer patients

Characteristic	CSS			OS		
	HR	95% CI	*P* value	HR	95% CI	*P* value
Year of diagnosis (continuous variable)	0.973	0.962–0.985	< 0.001	0.977	0.966–0.988	< 0.001
Age (years) (continuous variable)	1.006	1.001–1.011	0.028	1.014	1.009–1.019	< 0.001
Race						
Black	1			1		
White	0.907	0.731–1.127	0.379	0.882	0.721–1.073	0.209
Other/unknown	1.010	0.719–1.418	0.956	0.974	0.715–1.326	0.865
Sex						
Male	1			1		
Female	0.809	0.701–0.934	0.004	0.815	0.715–0.929	0.002
Histologic subtype						
Squamous	1			1		
Adenocarcinoma	0.997	0.882–1.127	0.958	0.986	0.882–1.102	0.803
Other	1.158	0.964–1.391	0.117	1.126	0.952–1.333	0.166
Tumor location						
Upper third	1			1		
Middle third	0.917	0.623–1.351	0.662	0.915	0.646–1.295	0.615
Lower third	0.757	0.523–1.094	0.138	0.738	0.530–1.028	0.073
Tumor stage						
T1	1			1		
T2	1.193	0.942–1.511	0.143	1.176	0.950–1.456	0.137
T3	1.600	1.320–1.940	< 0.001	1.513	1.270–1.801	< 0.001
T4	1.787	1.340–2.384	< 0.001	1.681	1.291–2.189	< 0.001
Nodal stage						
N0	1			1		
N1	1.731	1.538–1.948	< 0.001	1.562	1.400–1.741	< 0.001
N2	2.343	2.008–2.733	< 0.001	2.046	1.768–2.368	< 0.001
N3	3.256	2.578–4.112	< 0.001	2.771	2.211–3.474	< 0.001
Grade						
Well differentiated	1			1		
Moderately differentiated	1.183	0.897–1.565	0.239	1.211	0.939–1.563	0.141
Poorly/undifferentiated	1.527	1.159–2.012	0.003	1.496	1.163–1.924	0.002
LNR (continuous variable)	3.994	3.332–4.787	< 0.001	3.330	2.795–3.967	< 0.001
Number of RLNs (continuous variable)	0.989	0.984–0.995	< 0.001	0.988	0.983–0.993	< 0.001
Number of RLNs						
1–6	1			1		
7–10	1.046	0.914–1.196	0.515	0.978	0.864–1.107	0.723
11–17	0.876	0.764–0.997	0.045	0.847	0.750–0.957	0.007
18–71	0.765	0.660–0.886	< 0.001	0.747	0.653–0.854	< 0.001
Number of RLNs						
1–10	1			1		
11–71	0.809	0.730–0.895	< 0.001	0.809	0.737–0.888	< 0.001

CSS, cause-specific survival; OS, overall survival; HR, hazard ratio; CI, confidence interval; LNR, lymph node ratio; RLNs, resected lymph nodes.

The multivariate analysis incorporating covariates which were significant in the univariate analysis showed that an increasing number of RLNs was associated with better CSS and OS. Patients with 11–71 RLNs had better CSS (hazard ratio [HR] = 0.694, 95% confidence interval [CI]: 0.603–0.799, *P* < 0.001) and OS (HR = 0.724, 95% CI: 0.636–0.824, *P* < 0.001) than those with 1–10 RLNs. Other independent factors which affected CSS and OS were age, T stage, and N stage. However, LNR did not influence the prognosis (Table 3).

**Table 3:** Multivariate analysis of prognostic factors influencing the survival of esophageal cancer patients

Characteristic	CSS	95% CI	*P* value	OS	95% CI	*P* value
	HR			HR		
Year of diagnosis (continuous variable)	0.990	0.958–1.023	0.537	0.991	0.962–1.022	0.572
Age (years) (continuous variable)	1.013	0.005–1.020	0.001	1.018	1.011–1.025	< 0.001
Sex						
Male	1			1		
Female	0.950	0.785–1.149	0.597	0.956	0.803–1.138	0.614
Tumor stage						
T1	1			1		
T2	1.192	0.926–1.533	0.172	1.164	0.925–1.463	0.195
T3	1.365	1.104–1.687	0.004	1.370	1.131–1.659	0.001
T4	1.605	1.176–2.191	0.003	1.646	1.239–2.186	< 0.001
Nodal stage						
N0	1			1		
N1	1.851	1.577–2.172	< 0.001	1.708	1.474–1.980	< 0.001
N2	2.258	1.825–2.795	< 0.001	1.915	1.562–2.348	< 0.001
N3	3.763	2.783–5.088	< 0.001	3.159	2.356–4.235	< 0.001
Grade						
Well differentiated	1			1		
Moderately differentiated	0.929	0.660–1.306	0.573	0.947	0.692–1.295	0.732
Poorly/undifferentiated	1.104	0.788–1.545	0.566	1.062	0.779–1.448	0.702
LNR (continuous variable)	1.363	0.870–2.135	0.177	1.254	0.812–1.937	0.307
Number of RLNs						
1–10	1			1		
11–71	0.694	0.603–0.799	< 0.001	0.724	0.636–0.824	< 0.001

CSS, cause-specific survival; OS, overall survival; HR, hazard ratio; CI, confidence interval; LNR, lymph node ratio; RLNs, resected lymph nodes.

### Correlation of the number of RLNs and survival

**Figure 1:**
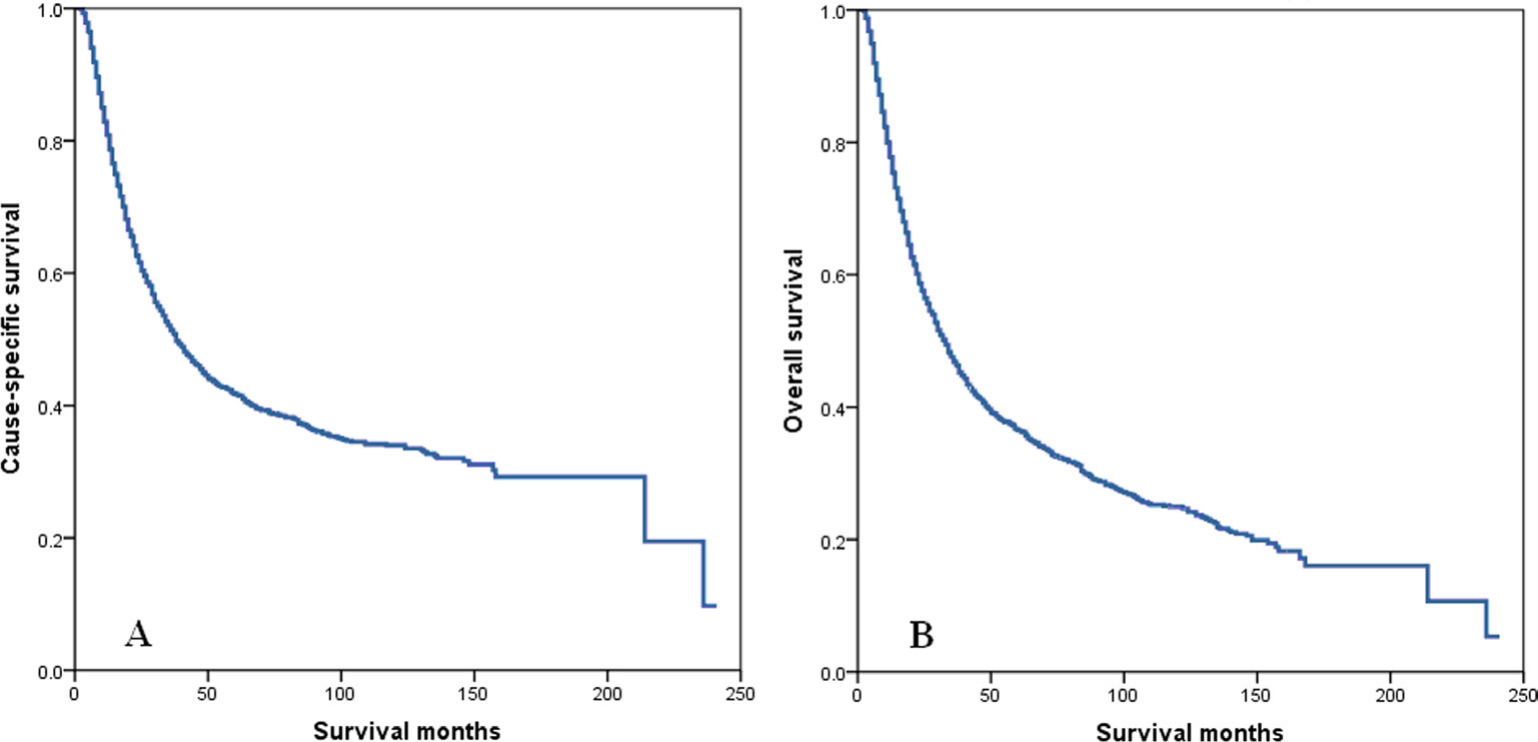
Cause-specific survival (A) and overall survival (B) of esophageal cancer patients with preoperative radiotherapy

**Figure 2:**
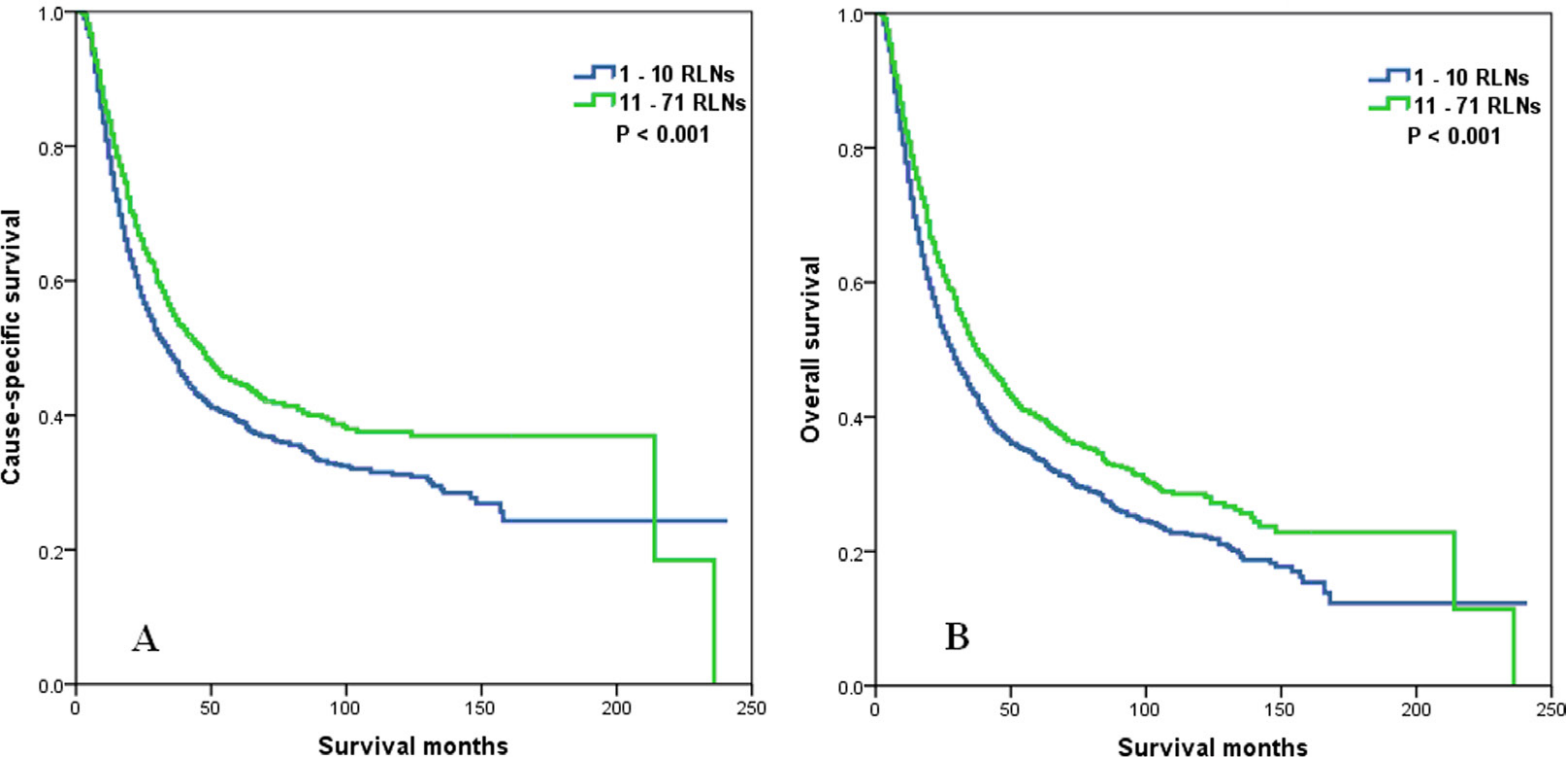
Cause-specific survival (A) and overall survival (B) of esophageal cancer patients with preoperative radiotherapy according to the number of resected lymph nodes

The median follow-up time of all patients was 21 months (range, 1–241 months), and 34 months (range, 1–241 months) in surviving patients. The 5-year CSS and OS were 41.8% and 36.5% (Figure 1A, 1B), respectively. The 5-year CSS was 39.1% and 44.8% in patients with 1–10 RLNs and 11–71 RLNs, respectively (*P* < 0.001) (Figure 2A). The 5-year OS was 33.7% and 39.9% in patients with 1–10 RLNs and 11–71 RLNs, respectively, and the median survival times were 28 and 38 months (*P* < 0.001) (Figure 2B).

**Figure 3:**
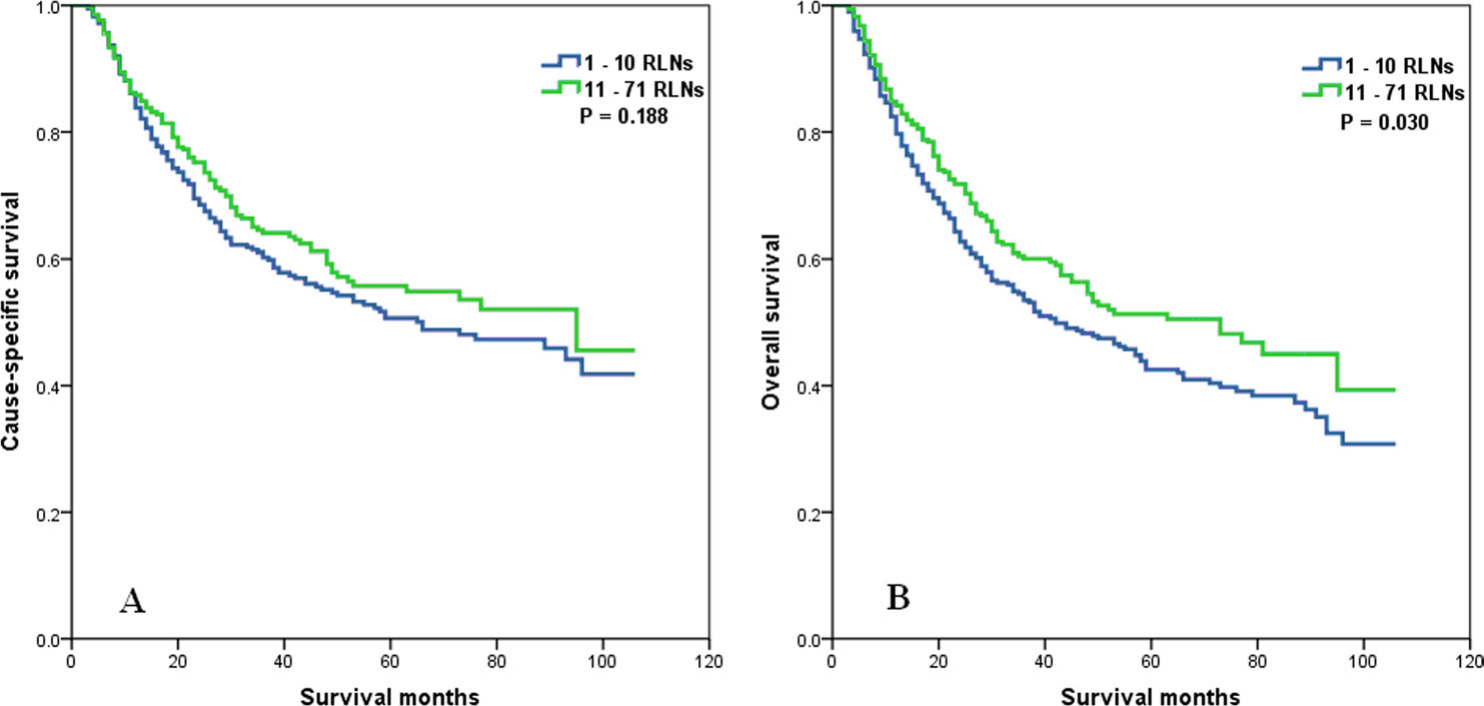
Cause-specific survival (A) and overall survival (B) of T1-2 stage esophageal cancer patients with preoperative radiotherapy according to the number of resected lymph nodes

**Figure 4:**
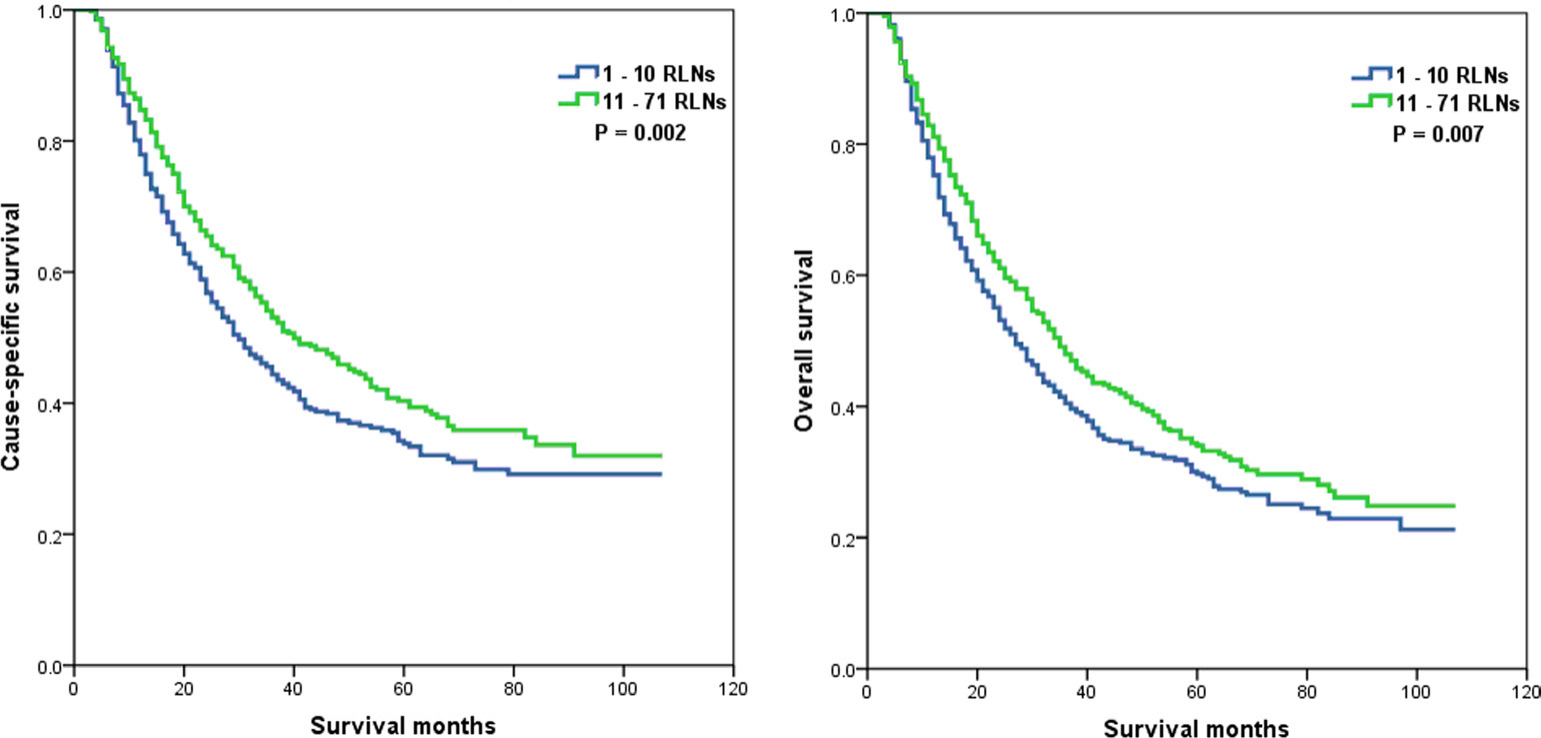
Cause-specific survival (A) and overall survival (B) of T3-4 stage esophageal cancer patients with preoperative radiotherapy according to the number of resected lymph nodes

Whether the effect of the number of RLNs on survival was modified by the T stage was determined. No association of the number of RLNs with CSS (*P* = 0.188) in patients with T1 or T2 stage was found, but the number of RLNs was significantly associated with OS (*P* = 0.030) (Figure 3A–3B). In patients with T3 or T4 stage disease, a higher number of RLNs was significantly associated with better CSS (*P* = 0.002) and OS (*P* = 0.007) (Figure 4A, 4B).

**Figure 5:**
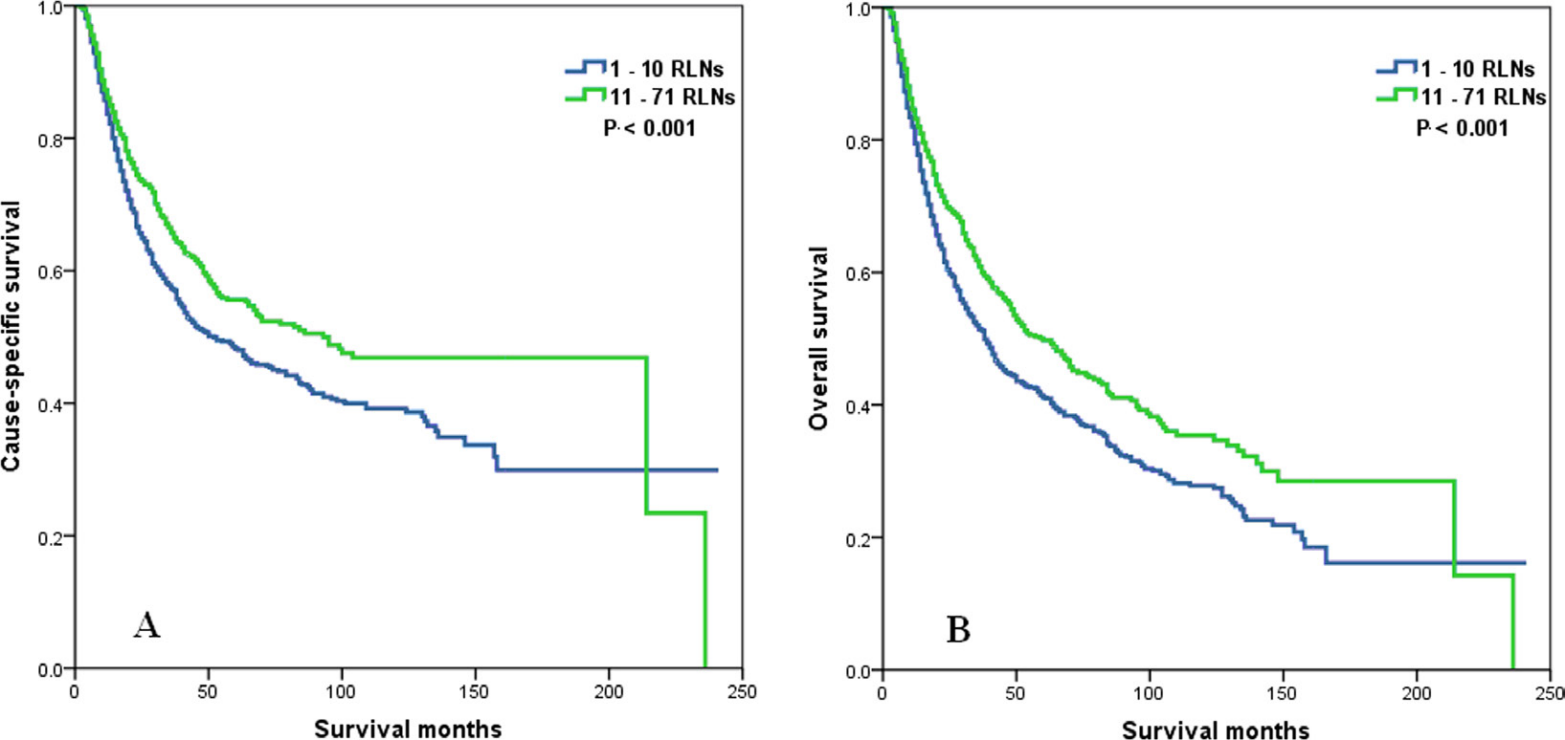
Cause-specific survival (A) and overall survival (B) of N0 stage esophageal cancer patients with preoperative radiotherapy according to the number of resected lymph nodes

**Figure 6:**
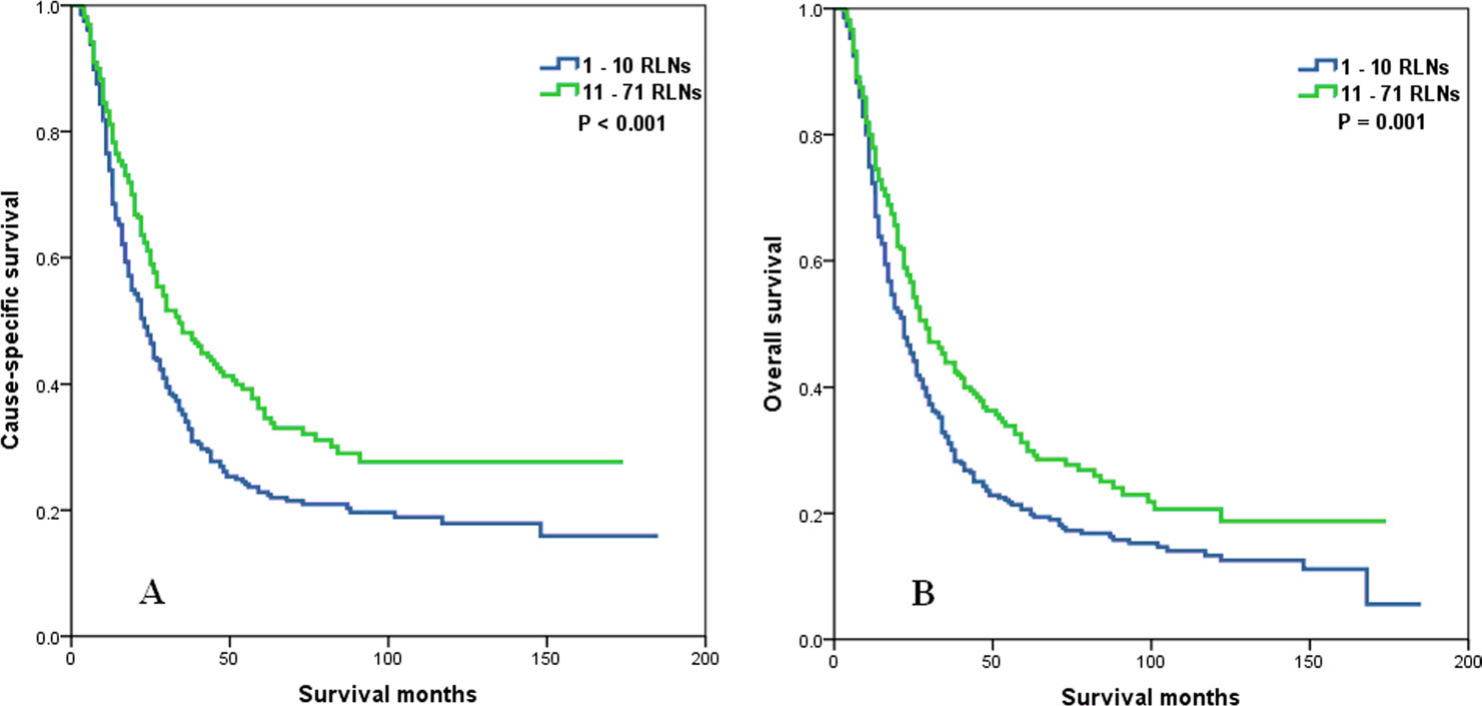
Cause-specific survival (A) and overall survival (B) of N1 stage esophageal cancer patients with preoperative radiotherapy according to the number of resected lymph nodes

**Figure 7:**
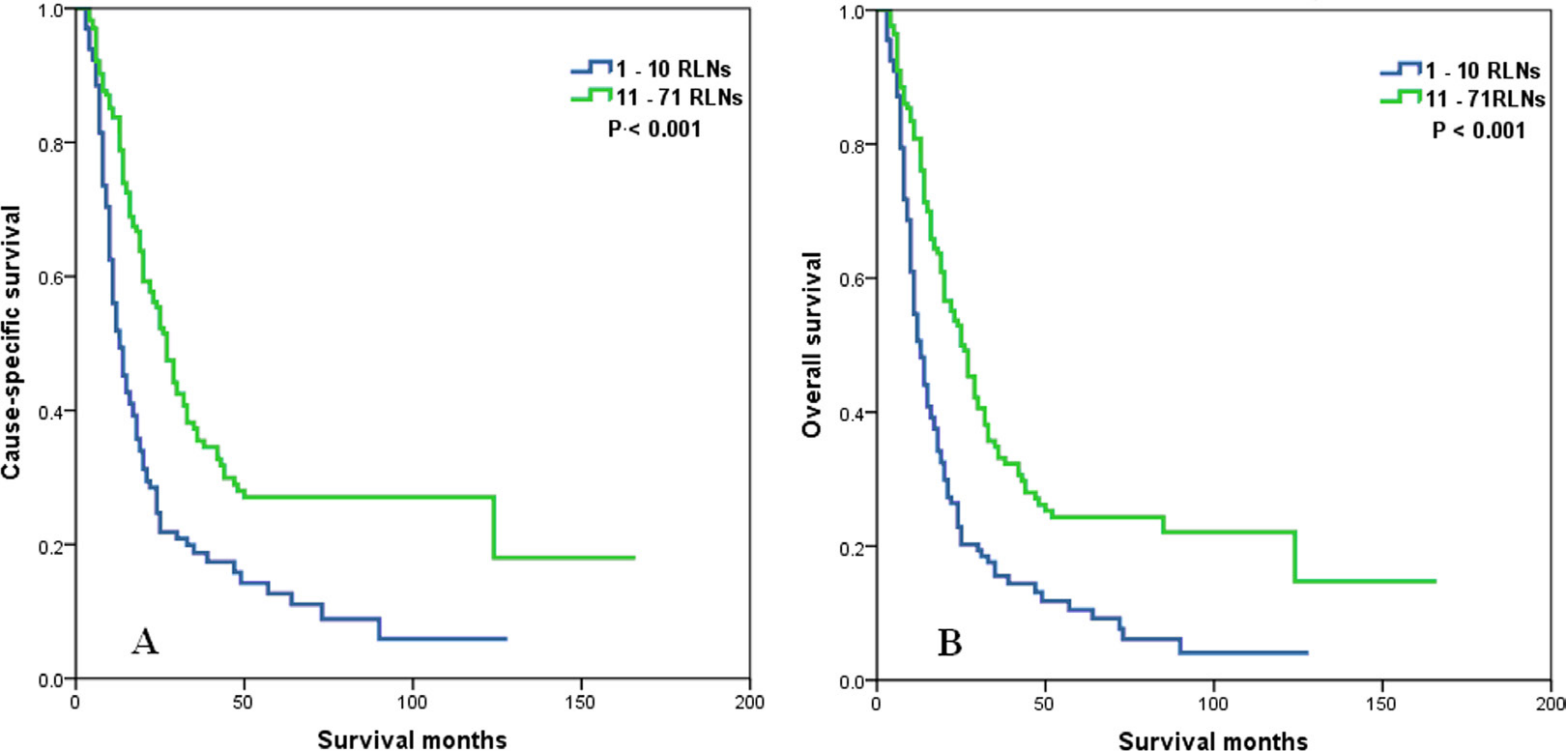
Cause-specific survival (A) and overall survival (B) of N2 stage esophageal cancer patients with preoperative radiotherapy according to the number of resected lymph nodes

**Figure 8:**
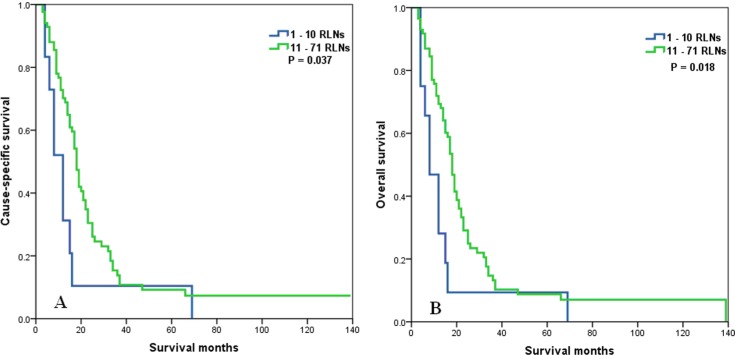
Cause-specific survival (A) and overall survival (B) of N3 stage esophageal cancer patients with preoperative radiotherapy according to the number of resected lymph nodes

The prognostic effect of the number of RLNs according to N stage was also examined. Patients with a higher number of RLNs significantly differed across N stage. In patients with N0, N1, N2, and N3 stage disease, a higher number of RLNs correlated with better CSS (*P* < 0.001 for N0 stage, *P* < 0.001 for N1 stage, *P* < 0.001 for N2 stage, and *P* = 0.037 for N3 stage) and OS (*P* < 0.001 for N0 stage, *P* = 0.001 for N1 stage, *P* < 0.001 for N2 stage, and *P* = 0.018 for N3 stage) (Figures 5–8).

## DISCUSSION

This study investigated the impact of the number of RLNs on survival after preoperative radiotherapy and cancer-directed surgery for EC patients using the SEER database. The results showed that the number of RLNs was an independent prognostic factor for CSS and OS, a higher number of RLNs was associated with better survival.

Studies have shown that nCRT could affect the number of lymph nodes harvested in colon cancer patients [14, 15]. However, it is still a matter of debate whether nCRT affects the number of RLNs in patients with EC. A phase III randomized controlled trial revealed that the number of RLNs in EC was lower in nCRT-treated patients than in non-nCRT-treated counterparts (16.0 vs. 22.0, *P* = 0.001) [13]. The randomized CROSS trial also found that the number of RLNs in patients with EC was significantly decreased after nCRT (18 vs.14, *P* < 0.001) [5]. However, a study by Luna et al. [16] showed that nCRT did not affect the number of RLNs (16.0 vs 15.5, *P* = 0.57). The current study also did not find that preoperative radiotherapy affected the number of RLNs. Although with the SEER database, we were unable to identify the preoperative staging of patients. However, according to our results, a greater number of RLNs in EC patients after nCRT could precisely stage the status of lymph node with preventing the stage migration, and better predict the prognosis of patients.

In this study, the number of positive lymph nodes is a prognostic factor in EC patients received neoadjuvant therapy, which is similar results from other studies [17, 18]. Therefore, an adequate number of resected lymph nodes is important for lymph node staging. For patients treated with surgery alone, the recommended number of RLNs varies with different pT stages [19]. However, the optimal number of RLNs in EC patients after neoadjuvant therapy has not been clearly defined. The CROSS trial indicated that the number of RLNs had no influence on the survival [5]. However, Hanna et al. [8] reported that patients with a higher number of RLNs had better survival. A study by Chao et al. [9] indicated that the RLN count was not impact the survival if a pathological complete response (PCR) was achieved after patients receiving nCRT, and a higher number of RLNs (≥ 8) was associated with better survival in patients without PCR. Another SEER study showed that patients with clinically node-positive disease should undergo both preoperative radiotherapy and adequate lymphadenectomy to ensure optimal survival [10]. Based on a large sample analyses, our results showed that CSS and OS were significantly better when the number of RLNs was more than 10. A possible explanation for this phenomenon is that the more number of lymph nodes resected, the more likely a patient will get better pathologic staging (they could be upstaged and received appropriate adjuvant therapy), thus improving locoregional control and possibly enhancing survival.

Tumor regression is an important indicator of nCRT, and the impact of nCRT on subsequent pT stage and nodal positivity requires further study. Stiles et al. performed a study following the guideline of the Worldwide Esophageal Cancer Collaboration (a minimum of 10 lymph nodes should be removed for pTis/T0/T1 cancers, 20 lymph nodes for pT2 cancers, and 30 lymph nodes for pT3/T4 cancers), and their results are still applicable for patients with neoadjuvant therapy [20]. In addition, after complete remission (pT0) of primary tumors in patients who receive preoperative radiotherapy, lymph node status is still a predictor of survival [21]. Our study demonstrated that the number of RLNs has prognostic value by T and N staging. Thus, it is suggested that surgeons should dissect as many lymph nodes as possible regardless of the therapeutic response.

There are limitations to the current study. This was a retrospective analysis, and the SEER database lacks of data on chemotherapy, co-morbidities, type of operation, chemotherapy regimens and dose, pathologic stage, and other data known to potentially influence survival such as performance status, institutional volume, and surgeon's volume. Therefore, it is hard to accurately evaluate the clinical condition of the patients. The primary strength of this study is the large number of patients available using the SEER registry, which may decrease the potential for selection and surveillance biases that are associated with single institution analysis. In addition, there is still no standard for the optimal number of RLNs in EC patients who received lymphadenectomy after neoadjuvant therapy. It is necessary to conduct a prospective multicenter study to verify the value of the number of RLNs in patients with EC, and investigate an optimal cut-off point of RLN count.

In conclusion, the number of RLNs was found to be an independent prognostic factor for EC patients who receive preoperative radiotherapy and cancer-directed surgery. The results suggest that as many lymph nodes as possible should be dissected to evaluate prognosis and guide treatment.

## PATIENTS AND METHODS

### Patients

Data was obtained from the SEER database (Surveillance Research Program, National Cancer Institute SEER*Stat software, http://www.seer.cancer.gov/seerstat) (Version 8.2.1), which consists of 18 population-based cancer registries. Permission was obtained to access research data files (reference number 11252-Nov2014) [22]. Patients with a diagnosis of EC who received preoperative radiotherapy and cancer-directed surgery from 1988 to 2012 were identified in the SEER database using the International Classification of Disease for Oncology, Third Edition. Data of EC patients who received CDS without preoperative radiotherapy were also collected to investigate the effect of radiotherapy on the number of lymph node removed. Patients with distant metastasis were excluded. Extraction of data from the SEER database does not require informed consent. This study was approved by the ethics committee of the First Affiliated Hospital of Xiamen University and Sun Yat-sen University Cancer Center.

### Clinicopathological factors

The following clinical and pathological factors were collected from the SEER database: year of diagnosis, race, age, sex, histological type, grade, tumor location, T stage, N stage, the number of RLNs, and the lymph node ratio (LNR). The number of RLNs was defined as the total number of regional lymph nodes that were removed as indicated in the SEER dataset. The LNR was defined as the ratio of the number of positive lymph node to the total number of RLNs. Vital status, cause of death, and the duration of follow-up were also recorded.

### Statistical analysis

The χ^2^ and Fisher's exact tests were used to analyze the differences between qualitative data. Univariate and multivariate Cox regression analyses were performed to analyze risk factors for cause-specific survival (CSS) and overall survival (OS). Multivariable analyses were performed for factors which were significantly associated with CSS and OS in univariate analyses. Survival rates were plotted by the Kaplan-Meier method, and compared using the log-rank test. All data were analyzed with the SPSS statistical software package, version 21.0 (IBM Corporation, Armonk, NY, USA). A value of *P* < 0.05 was considered statistically significant.
